# Ductal Carcinoma In Situ of the Breast: Perspectives on Tumor Subtype and Treatment

**DOI:** 10.1155/2020/7251431

**Published:** 2020-05-27

**Authors:** Yufei Liu, Kangquan Shou, Juanjuan Li, Qi Wu, Yuchang Hu, Junjie Wang, Chunyu Cao, Qing Wang

**Affiliations:** ^1^Institute of Pathology, China Three Gorges University, Yichang 443000, China; ^2^Department of Pathology, Yichang Central People's Hospital, Yichang 443003, China; ^3^Department of Orthopaedics, Yichang Central People's Hospital, Yichang 443003, China; ^4^Department of Breast and Thyroid Surgery, Renming Hospital of Wuhan University, Wuhan 430060, China; ^5^Department of Obstetrics and Gynecology, Ren He Hospital of China Three Gorges University, Yichang 443001, China; ^6^Hubei Key Laboratory of Tumor Microenvironment and immunotherapy, China Three Gorges University, Yichang 443000, China

## Abstract

**Objective:**

To evaluate ductal carcinoma in situ (DCIS) characteristics and the effect of different treatment strategies. *Patients and Methods*. Using data with known hormone receptor (HoR) and human epidermal growth factor receptor 2 (HER2) status obtained by the Surveillance, Epidemiology, and End Results (SEER) program from 2010-2014, the study was conducted to investigate tumor subtype-specific differences in various characteristics, overall survival (OS), and breast cancer-specific mortality (BCSM).

**Results:**

A total of 3415 patients with DCIS were eligible. Compared with HoR+/HER- subgroup, patients with triple-negative (TN) and HoR-/HER+ were commonly higher in grade, larger in size, and tended to receive mastectomy (*P* < 0.05). The multivariate analysis revealed that patients with TN were more likely to have a poorer OS and show a higher breast cancer-specific mortality compared with the HoR+/HER- subgroup (*P* < 0.05). Multivariate analysis on the history of local treatment and surgery showed patients receiving breast-conserving surgery (BCS) plus radiotherapy (R) and BCS plus axillary lymph node dissection was likely to improve OS without affecting breast cancer-specific mortality (*P* < 0.05).

**Conclusion:**

The results demonstrate that DCIS associated with TN subtype portends poor prognosis. Meanwhile, BCS plus R was a preferable option and resulted in survival rates better than those achieved with mastectomy, and SLNB should be considered as an appropriate assessment of axillary staging in patients with DCIS.

## 1. Introduction

Ductal carcinoma in situ (DCIS) of the breast describes lesions comprised of abnormal epithelial cells that are completely confined within breast ducts without invasion beyond the basement membrane. In the majority of patients, DCIS is primarily viewed as the indicator of invasive breast cancer (IBC) [1, 2]. Notably, the positive detection rate of DCIS increased rapidly after the introduction of mammography as a population screening tool and substantially increased at a slower rate [3–5].

As previously mentioned, DCIS has been characterized as a type of breast cancer with small in size, low grade, and estrogen receptor (ER) and progesterone receptor (PR) positive expression, but HER2 testing is not a routine part of the pathologic evaluation [6, 7]. However, studies suggested that DCIS lesions with high nuclear grade are often ER negative and HER2 overexpression. Furthermore, HER2 overexpression has also been demonstrated to be associated with the reduction of survival, and targeting HER2 has been a potential treatment strategy for HER2-overexpressing DCIS.

The traditional treatment of DCIS is mastectomy, whereas breast conservation surgery (BCS) is a feasible surgical option for selected patients. In addition to BCS, outcomes related to axillary lymph node dissection in DCIS have not been validated in well-controlled clinical trials. Based on the current guideline, SLNB has been recommended as a less invasive method to stage patients with early invasive ductal carcinoma than axillary lymph node dissection (ALND), but routine use of SLNB for DCIS is not supported. We speculate that SLNB would be approved as an effective method in detecting the status of axillary lymph nodes (ALNs). The benefit of radiotherapy, in terms of a significantly reduced risk of local recurrence in those undergoing BCS, has been demonstrated by several large randomized controlled trials [8, 9]. However, it is not clear whether radiotherapy should be appropriate in selected low-risk cases. The clinical characteristics of DCIS and optimal treatment strategy are topics of uncertainty and concern for both patients and clinicians. Therefore, this article aims at evaluating DCIS characteristics and the effect of different treatment strategies, with special emphasis on the role of subtypes of DCIS, BCS, and SLNB.

## 2. Patients and Methods

### 2.1. Patient Selection

All the patient data were from the National Cancer Institute's Surveillance, Epidemiology, and End Results (SEER) program between 2010 and 2014. 2010 was selected as the starting point of our measurements as SEER began HER2 status collection in 2010. We had used the *International Classification of Diseases for Oncology*, 3rd edition (ICD-O-3) histopathology codes to extract all cases with DCIS (codes 8500, 8523), basing on a scheme used previously [10]. The selected cases were all with known breast subtypes. Cases that did not receive surgery, for which the histologic types were unknown, or were diagnosed at autopsy were excluded. Demographic variables included age at diagnosis (<35, 35–49, 50–64, and >65 years). Cancer characteristics were classified by grade (well, moderately, poorly, undifferentiated, and unknown), tumor size (≤10, 10-20, 20-50, and >50 mm), and laterality (right, left, others, and unknown). Treatment characteristics included receipt of radiation therapy (no, yes, and unknown). All the subtypes were characterized according to the breast subtype variable as HoR+/HER2-, HoR+/HER2+, HoR-/HER2+, and triple-negative (TN). Patients were categorized as receiving BCS (surgery of primary site variable values of 20–24) and mastectomy (surgery of primary site variable values of 30–80). Because the type of axillary surgery was not reported within SEER, replacement of 1–5 lymph nodes removed was regarded as axillary lymph node dissection groups and >5 lymph nodes removed was taken for axillary lymph node dissection (ALND) groups, as shown in previous studies [11]. We characterized several subgroups named “local treatment” (mastectomy (M), BCS only, and BCS with radiotherapy (BCS+R) and “Surgery” (M+ALND, M+SLNB, BCS+ALND, BCS+SLNB) (Table 1). Specifically, the radiotherapy is the adjuvant therapy only after breast-conserving surgery, not for the patients who received mastectomy without axillary lymph node dissection. And the radiotherapy is limited to the whole breast not including the axillary area.

The two primary outcomes in our study were OS and breast cancer-specific mortality. Vitality status was recorded as “alive” or “dead” in the SEER dataset. Survival time (in months) was calculated for each patient using the “Completed Months of Follow-up” in the SEER database. OS was determined by patients who were alive at the end of the study period or who were alive at their last follow-up. Breast cancer-specific mortality was determined by patients whose cause of death was due to breast cancer with patients who were alive at the end of the study period, had died due to other causes, or who were alive at their last follow-up. Cases without survival times were classified as unknown and removed from this study.

### 2.2. Statistical Analysis

Patient demographics and cancer-related characteristics were compared within subgroups using Chi-square or Fisher's exact tests. Survival outcomes on OS and breast cancer-specific mortality were estimated using the weighted Kaplan-Meier method, and variables were compared using the log-rank test in the subgroups. Univariate and multivariate Cox proportional hazard regressions were used to obtain hazard ratios (HRs) and their respective 95% confidence intervals and show the strength of the estimated relative risk; these approaches were applied to model the relationship between potential covariates and either OS or breast cancer-specific mortality. All statistical analyses and all charts of survival probabilities were performed using SPSS 19.0 (IBM Corporation, Armonk, NY). A two-sided *P* value < 0.05 was considered statistically significant.

## 3. Results

A total of 3415 patients with DCIS were eligible during the study period from 2010 to 2014. A total of 2050 DCIS cases within HoR+/HER2- subgroup, 765 in HoR+/HER2+, 365 in HoR-/HER2+, and 235 TN (triple negative) patients had available information and were included within this study.

Differences in patient demographics, cancer characteristics, treatments, and outcomes within subgroups were summarized in Table 2. Compared with HoR+/HER2- subgroup, patients with TN were commonly higher in grade, larger in size, and older in age (each *P* < 0.05). Patients within HoR-/HER2+ subgroup were more universally higher in grade and larger in size compared with HoR+/HER2- subgroup. For treatment options, patients with TN and HoR-/HER2+ subgroup tended to receive mastectomy, and all cases were increasingly treated by SLNB.

**Figure 1:**
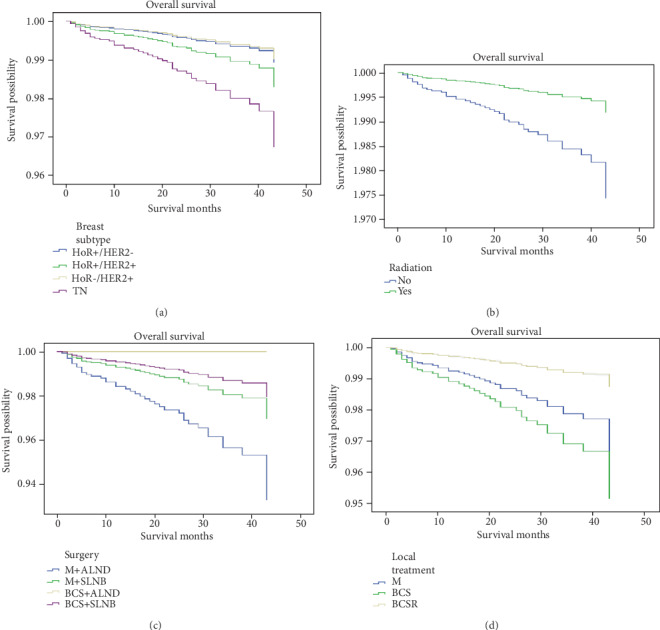
Weighted Kaplan-Meier curves of overall survival (OS) in subgroup analysis. (a) OS is based on breast subtype. (b) OS is based on radiotherapy. (c) OS is based on surgery. (d) OS is based on local treatment.

**Figure 2:**
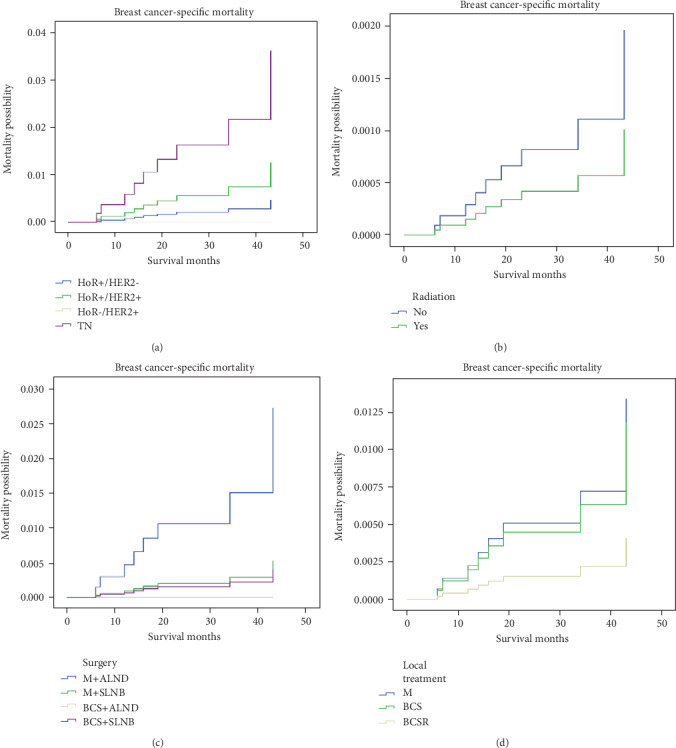
Weighted Kaplan-Meier curves of breast cancer-specific mortality (BCSM) in subgroup analysis. (a) BCSM is based on breast subtype. (b) BCSM is based on radiotherapy. (c) BCSM is based on surgery. (d) BCSM is based on local treatment.

Weighted Kaplan-Meier analysis was used to determine OS and breast cancer-specific mortality in the subgroups based on breast subtype. Survival curves for the subgroups were generated (Figures 1(a) and 2(a)). At the median follow up 42 months, patients with TN had an OS of 97.0% compared with 98.6% in the HoR+/HER2- subgroup (*P* < 0.05). Further, the breast cancer-specific mortality rate was 1.5% for the TN group compared with 0.2% for the HoR+/HER2- subgroup (*P* < 0.05). We used multivariate analysis based on the weighted Kaplan-Meier results. All the prognostic factors that predicted OS and breast cancer-specific mortality were analyzed on multivariate analysis (Table 2). In the multivariate analysis, patients with TN were more likely to have a poorer OS and a higher breast cancer-specific mortality were showed compared with the HoR+/HER2- subgroup (OS, *P* = 0.01, adjusted hazard ratio (aHR) = 3.092; breast cancer-specific mortality, *P* = 0.012, aHR = 7.725).

During the follow-up period, we analyzed adjuvant radiotherapy, showing that patients undergoing radiotherapy showed a higher OS without affecting breast cancer-specific mortality (Figures 1(b) and 2(b), OS, *P* = 0.004, aHR = 0.314; breast cancer-specific mortality, *P* = 0.35, aHR = 0.514).

Furthermore, our findings demonstrated that compared with the patients treated with M+ALND, those patients underwent with BCS+SLNB were likely to display a better OS (Figures 1(c) and 2(c), *P* = 0.014, aHR = 0.298). Additionally, multivariate analysis of OS on history of local treatment showed patients receiving BCS combined with radiotherapy (R) was likely to improve OS compared with those in the mastectomy subgroup (*P* = 0.016, aHR = 0.374), but did not affect breast cancer-specific mortality (Figures 1(d) and 2(d)).

## 4. Discussion

In our previous work [11], we found that a poorer survival in TN subtype when adjusting for other factors based on a large population-based cohort of cases diagnosed with breast carcinoma in situ (BCIS), furthermore, in the current study, with a longer follow-up duration (median follow-up, 42 months), we found similar results in a large series of DCIS histological subtypes. Furthermore, we focus on DCIS. In our series, there was a dramatic difference within the subgroups of local treatment, radiotherapy, and surgical strategy only for OS.

In previous studies, a new Van Nuys Prognostic Index (VNPI) is used as the independent predictor of local recurrence, which derives a new formula, including tumor size, margin width, pathologic grade, and age [12]. However, our research showed that tumor size, nuclear grade, and age were not the prognostic factors for OS or breast cancer-specific mortality. In the current study, patients within the HoR-/HER2+ subgroup were higher in grade and larger in size compared with HoR+/HER2- subgroup. The correlation between HER2 and tumor behavior has been described previously. That study revealed that HER2 was overexpressed in 24/31 (77%) patients who experienced local relapse for DCIS [13]. Whereas, another study showed that HER2 overexpression may not be the key factor in the progression of DCIS transforming to invasive carcinoma, and HER2 gene amplification is inversely related to the invasive progression in DCIS patients [14, 15]. Simultaneously, the precise incidence of HER2 overexpression in many cases of DCIS is unclear. A retrospective analysis showed that HER2 was overexpressed in 61% of cases with DCIS [16]. In contrast, Roses et al. [17] reported 106 patients with DCIS noted HER2 overexpression in only 37% of cases. Furthermore, they also described an association between HER2 overexpression and the detection of invasive foci on surgical specimens. However, we analyzed that HER2 status was not correlated with survival adjusting for other prognostic factors. This conclusion remains to be further confirmed by prospective trial.

The TN subtype of DCIS was diagnosed seldom, possibly because the tumor progression of TN subtype is quicker than other types of breast cancer. In consistent with the previous study, our study reported that the TN subtype may be much faster in progression than other breast cancer types [15]. In this study, data showed that patients with TN subtype tended to be higher in grade, larger in size, and older in age, and the prognosis of TN subtype were more likely to be poor. Some reports had suggested that TN subtype DCIS may be a potential precursor to TN invasive cancer [18, 19], and a more frequent and rapid progression from DCIS to invasive cancer was related to the comedo subtype of DCIS [20, 21]. Consequently, some unrecognized mechanisms or features helping TN subtype in progression should be further studied.

Currently, the treatment strategy of DCIS was controversial. Although mastectomy was curative for approximately all of patients with DCIS [22, 23], mastectomy is still considered as overtreatment. Although radiotherapy has been proved to decrease local recurrence compared with BCS alone [24], it may also lead to overtreatment for the low-risk patients. Local excision only was regarded as an appropriate surgery for patients with low-risk DCIS in several studies [12, 25]. Furthermore, several retrospective studies [26–30] reported that BCS resulted in consistently higher rates of local recurrence (range, 8%–34%) compared with patients treated by BCS+R (range, 0%–17%). Similarly, our results also presented that patients receiving BCS+R was likely to improve OS compared with those in the mastectomy subgroup. Therefore, BCS combined with radiotherapy was a preferable alternative for patients with DCIS. According to the previous study, axillary metastases were found in approximately 1-2% of DCIS cases [31], therefore, the need for axillary staging still remains necessary [32, 33]. Previously, SLNB was considered feasible for patients with DCIS [34–36]. In our study, patients undergoing BCS+SLNB showed better survival than those receiving M+ALND, which can be attributed to the radiotherapy. However, routine use of SLNB is only recommended for high-risk DCIS [35].

About the molecular profiles of DCIS, some studies had confirmed that DCIS was high expression of estrogen receptor (ER) associated with low-grade lesions, but positive for c-erbB-2 (HER2), Ki67, and p53 associated with high-grade lesions [31, 37, 38]. A recent case–control study suggested that DCIS cases with triple positive for p16, COX-2, and Ki67 had a significantly higher rate of progression to invasive breast cancer than those of negative for these biomarkers (8-year risks of subsequent invasive cancer 19.6% and 4.1%, respectively) [39]. Simultaneously, several studies confirmed that HoR negativity, high S-phase fraction, abnormal DNA ploidy, P53 overexpression, and HER2 over-expression were associated with more aggressive tumor behavior in DCIS [40–43]. Gene expression profiling was likely to enhance our understanding on DCIS behavior and its relation to invasive breast cancer. The findings of several studies [40, 44] had recorded differential expression patterns and identified new facets of the earliest stage of breast cancer progression. More molecular and genetic studies that predict local recurrence and progression to invasive breast cancer independent of standard prognostic markers would be required, and the difference in survival must continue to be monitored.

The main limitations of our study were heterogeneous population and retrospective setting. Furthermore, the information on systemic therapy and margin control was insufficient. In doing so, HER2 targeted therapy and novel adjuvant hormone therapy should remain utilized for the management of DCIS to improve survival. Additionally, we had no specific information on the type of axillary operation, and thus used the number of lymph nodes removed as a surrogate of the type of axillary surgery (SLNB vs. ALND).

## 5. Conclusions

Despite the limitations listed above, our study demonstrated that DCIS appears to alter prognosis associated with TN subtype. Meanwhile, BCS plus R was a preferable option and resulted in survival rates better than those achieved with mastectomy, and SLNB should be considered as an appropriate assessment of axillary staging in patients with DCIS. However, the surgical treatment plan must be chosen for the strength of helpful clinical and imaging assessment. Further researches are needed to minimize variation in modes of treatment and establish a standardized management approach.

## Figures and Tables

**Table 1:** Patient characteristics within subgroups.

Variables	HoR+/HER- *N* = 2050 (%)	HoR+/HER+ *N* = 765 (%)	HoR-/HER+ *N* = 365 (%)	TN *N* = 235 (%)	*P* value^∗^
Follow-up (months)	28.47 ± 17.29	28.12 ± 17.72	27.50 ± 17.03	30.48 ± 17.45	
Age at diagnosis (y)					*P* < 0.001
<35	11 (0.5)	9 (1.2)	3 (0.8)	0 (0.0)	
35-49	417 (20.3)	173 (22.6)	68 (18.6)	36 (15.3)	
50-64	808 (39.4)	346 (45.2)	171 (46.8)	108 (46.0)	
≥65	814 (39.7)	237 (31.0)	123 (33.7)	91 (38.7)	
Grade					*P* < 0.001
Well	363 (17.7)	39 (5.1)	5 (1.4)	7 (3.0)	
Moderately	810 (39.5)	193 (25.2)	38 (10.4)	45 (19.1)	
Poorly	553 (27.0)	406 (53.1)	258 (70.7)	143 (60.9)	
Undifferentiated	55 (2.7)	35 (4.6)	21 (5.8)	12 (5.1)	
Unknown	269 (13.1)	92 (12.0)	43 (11.8)	28 (11.9)	
Tumor size (mm)					*P* < 0.001
≤10	847 (41.3)	278 (36.3)	109 (29.9)	73 (31.1)	
10-20	396 (19.3)	179 (23.4)	89 (24.4)	69 (29.4)	
20-50	209 (10.2)	103 (13.5)	64 (17.5)	28 (11.9)	
>50	60 (2.9)	29 (3.8)	17 (4.7)	7 (3.0)	
Unknown	538 (26.2)	176 (23.0)	86 (23.6)	58 (24.7)	
Laterality					0.254
Left	1051 (51.3)	388 (50.7)	201 (55.1)	110 (46.8)	
Right	999 (48.7)	377 (49.3)	164 (44.9)	125 (53.2)	
Radiotherapy					0.480
None/unknown	1052 (51.3)	380 (49.7)	183 (50.1)	130 (55.3)	
Yes	998 (48.7)	385 (50.3)	182 (49.9)	105 (44.7)	
Local treatment					0.009
M	574 (28.0)	252 (32.9)	126 (34.5)	90 (38.3)	
BCS	1402 (68.4)	489 (63.9)	222 (60.8)	140 (59.6)	
None	69 (3.4)	21 (2.7)	15 (4.1)	4 (1.7)	
Unknown	5 (0.2)	3 (0.4)	2 (0.5)	1 (0.4)	
Axillary treatment					*P* < 0.001
None	1285 (62.7)	432 (56.5)	188 (51.5)	121 (51.5)	
SLNB	663 (32.3)	290 (37.9)	152 (41.6)	101 (43.0)	
ALND	84 (4.1)	37 (4.8)	23 (6.3)	12 (5.1)	
Unknown	18 (0.9)	6 (0.8)	2 (0.5)	1 (0.4)	
Status					*P* < 0.001
Alive	1998 (97.5)	745 (97.4)	356 (97.5)	218 (92.8)	
Dead	52 (2.5)	20 (2.6)	9 (2.5)	17 (7.2)	
Breast cancer	5 (0.2)	3 (0.4)	2 (0.5)	6 (2.6)	
Other	47 (2.3)	17 (2.2)	7 (1.9)	11 (4.7)	

^∗^ *P* values calculated by Pearson Chi -squared testing; bold if statistically significant, *P* < 0.05. y: years; mm: millimeter; y: years; BCS: breast-conserving surgery; HoR: hormone receptor; TN: triple negative; M: mastectomy; SLNB: sentinel lymph node biopsy; ALND: axillary lymph node dissection.

**Table 2:** Cox proportional hazards regression model analysis of overall survival (OS) and breast cancer-specific mortality (BCSM).

Variables	OS		BCSM	
	aHR (95% CI)	*P* value	aHR (95% CI)	*P* value
Age at diagnosis (y)				
<35	Reference		Reference	
35-49	564.1 (0.0, 1.49E58)	0.922	5.092 (0.0, 1.58E177)	0.994
50-64	550.8 (0.0, 1.45E58)	0.923	1591.7 (0.0, 3.37E179)	0.972
≥65	3134.5 (0.0, 8.24E58)	0.902	8686.1 (0.0, 1.84E180)	0.965
Grade				
Well	Reference		Reference	
Moderately	4.965 (0.647, 38.096)	0.123	2275.2 (0.0, 3.711E22)	0.732
Poorly	1.318 (0.158, 11.018)	0.799	3735.7 (0.0, 6.985E22)	0.716
Undifferentiated	2.882 (0.243, 34.136)	0.401	69.918 (0.0, 1.380E30	0.898
Laterality				
Left	Reference		Reference	
Right	1.913 (0.983, 3.724)	0.056	14.934 (0.685, 325.57)	0.086
Tumor size (mm)				
≤10	Reference		Reference	
10-20	1.411 (0.602, 3.307)	0.428	0.003 (0.0, 2405831.4)	0.579
20-50	0.633 (0.172, 2.338)	0.493	0.138 (0.003, 7.196)	0.327
>50	1.750 (0.445, 6.888)	0.423	0.267 (0.009, 7.787)	0.443
Subtype				
HoR+/HER-	Reference		Reference	
HoR+/HER+	1.269 (0.480, 3.354)	0.630	1.595 (0.083, 13.188)	30.732
HoR-/HER+	2.518 (0.2918, 6.909)	0.073	0.365 (0.007, 17.853)	0.611
TN	3.878 (1.619, 9.289)	0.002	17.093 (0.467, 625.28)	0.122
Radiotherapy				
No	Reference		Reference	
Yes	0.261 (0.083, 0.891)	0.021	3.751 (0.110, 127.66)	0.463
Local treatment				
M	Reference		Reference	
BCS	1.274 (0.512, 3.171)	0602	0.0 (0.0, 4.739E42)	0.886
BCS+R	0.270 (0.101, 0.718)	0.009	0.001 (0.0, 1.492E18)	0.764
Surgery				
M+ALND	Reference		Reference	
M+SLNB	0.5484 (0.236, 1.275)	0.163	0.014 (0.001, 0.389)	0.012
BCS+ALND	0.891 (0.170, 4.666)	0.891	0.0 (0.0)	0.984
BCS+SLNB	0.265 (0.099, 0.705)	0.008	0.0 (0.0, 2.337E178)	0.945

^∗^ *P* values calculated by Log-rank testing; bold if statistically significant, *P* < 0.05. M: mastectomy; BCS: breast-conserving surgery; R: radiotherapy; HoR: hormone receptor; TN: triple negative; LN: lymph node; SLNB: sentinel lymph node biopsy; ALND: axillary lymph node dissection. aHR: adjusted hazard ratio (adjusted for age at diagnosis, race, grade, tumor size, laterality, ER, PR, HER2, subtype, radiotherapy, local treatment, and surgery).

## Data Availability

This is an open access article distributed under the Creative Commons Attribution License, which permits unrestricted use, distribution, and reproduction in any medium, provided the original work is properly cited.

## Abbreviation

DCIS: Ductal carcinoma in situ
IBC: Invasive breast cancer
ER: Estrogen receptor
PR: Progesterone receptor
BCS: Breast conservation surgery
ALND: Axillary lymph node dissection
SEER: Surveillance, epidemiology, and end results
HRs: Hazard ratios.
